# *Bmp9* modulates cell proliferation and intercellular junctions in HERS during tooth root development

**DOI:** 10.1016/j.gendis.2025.101777

**Published:** 2025-07-23

**Authors:** Chang Liu, Hongyan Yuan, Jindie Huang, Shidian Ran, Xiaorui Wei, Xingrui Yan, Linyu Xue, Tong-Chuan He, Yuxin Zhang, Mengqin Gu, Si Wu, Fugui Zhang, Wenping Luo, Hongmei Zhang

**Affiliations:** a Chongqing Key Laboratory for Oral Diseases, The Affiliated Hospital of Stomatology of Chongqing Medical University, Chongqing 401147, China; b Department of Pediatric Dentistry, The Affiliated Stomatology Hospital, Chongqing Medical University, Chongqing 401147, China; c Chongqing Municipal Key Laboratory of Oral Biomedical Engineering of Higher Education, Chongqing Municipal Health Commission Key Laboratory of Oral Biomedical Engineering, Chongqing 401147, China; d Laboratory Animal Center of Southwest University, Chongqing 400799, China; e Molecular Oncology Laboratory, Department of Orthopaedic Surgery and Rehabilitation Medicine, The University of Chicago Medical Center, Chicago, IL 60637, USA

**Keywords:** Bone morphogenetic protein 9, Dentinogenesis, Hertwig's epithelial root sheath, Odontoblastic differentiation, Tooth root development

## Abstract

Tooth formation is a highly orchestrated process that precisely regulates the size and shape of the tooth. During typical tooth development, Hertwig's epithelial root sheath (HERS) interacts with mesenchymal cells to direct the elongation of the tooth root and the deposition of dentin and cementum, thereby contributing to the formation of a fully developed tooth root. BMP9, a member of the BMP family, plays a significant role in growth, development, and cell differentiation. However, the precise function of BMP9 in dental root development remains unclear, particularly regarding its influence on HERS and odontoblasts. In this study, we utilized a mouse molar model to investigate the role of BMP9 signaling in tooth root development. The tooth formation of *Bmp9* knockout (*Bmp9*-KO) mice and wild-type (WT) littermates was compared. Our findings revealed that *Bmp9*-KO mice exhibited shorter mandibular first molar roots, wider apical foramina, and thinner dentin compared with WT mice by micro-CT and hematoxylin-eosin staining analysis. Additionally, the results of immunohistochemistry and quantitative PCR indicated that in the absence of *Bmp9*, odontoblast differentiation and secretory function were compromised. Furthermore, *Bmp9* ablation resulted in reduced cell proliferation and increased intercellular junctions within HERS, subsequently impacting root dentin formation and apical foramen closure. This study offers new insights into the regulatory role of BMP9 signaling in odontoblast and HERS function, highlighting its significance in root development and providing potential avenues for future research in tooth root regeneration.

## Introduction

The root is the portion of the tooth that is embedded in the alveolar bone and fixed within the alveolar socket, providing vital support. The interaction between Hertwig's epithelial root sheath (HERS) and mesenchymal cells is the initiating factor for root dentin formation. Any abnormality in the HERS during tooth root formation can lead to abnormal development of the tooth root.^1^ On postnatal day 7, the development of the mandibular first molar tooth germ enters the mineralization stage, and HERS begins to form, marking the beginning of root development. A complex process influenced by HERS and mesenchyme initiates and promotes root formation.^2^^,^^3^ Upon contact with the basement membrane on the inner side of the HERS, dental papilla cells differentiate into odontoblasts.^4^

Research has demonstrated that HERS significantly contributes to odontoblast differentiation and influences the size, shape, and number of tooth roots.^5^, ^6^, ^7^ The conditional knockdown of *β-catenin* or transforming growth factor-β receptor 2 (*Tgf-βR2*) specifically in odontoblasts impaired the differentiation of root odontoblasts and disrupted the formation of HERS, resulting in abnormal root development in molars.^8^ Odontoblasts express dentin sialophosphoprotein (*Dspp*) at high levels, which is essential for proper dentin formation.^9^^,^^10^ Furthermore, modifications in cell proliferation and apoptosis within HERS are closely linked to the morphological transitions observed during root development. TGF-β/BMP/SMAD regulates sonic hedgehog (*Shh*) secretion from HERS, controlling mesenchymal nuclear factor I–C (Nfic) expression and root elongation. HERS plays a central role in the orchestration of epithelial-mesenchymal interactions.^11^ Nevertheless, further investigation is needed to understand how HERS controls root development.

Bone morphogenetic protein 9 (BMP9), also referred to as growth differentiation factor 2 (GDF2), belongs to the BMP family^12^ and is thought to participate in multiple physiological and pathological processes in oral biology, including jawbone formation,^13^^,^^14^ condylar cartilage remodeling,^15^ muscle regeneration,^16^ vascular malformations,^17^^,^^18^ salivary gland fibrosis,^19^ and tumorigenesis.^20^ Recent literature also reports that many BMP family signaling molecules play important regulatory roles in tooth development: Up-regulation of *Bmp6* expression is the basis for a significant increase in the number of teeth^21^; knockout of *Bmp2* and *Bmp4* in mouse epithelium shortens tooth roots.^22^ BMP9 signaling can activate the Wnt/β-catenin signaling pathway, thereby affecting downstream target gene transcription and regulating cell proliferation and differentiation.^23^ Our previous studies have shown that *Bmp9* was crucial for tooth and alveolar bone formation. In *Bmp9*-KO mice, the tooth cusps exhibited significant abrasion, along with shorter roots and decreased dentin thickness.^24^ Collectively, these findings indicate that BMP9 signaling likely regulates tooth root development. Therefore, it remains unclear whether *Bmp9* modulates tooth root development by regulating the development of odontoblasts and HERS.

In this study, we demonstrate that the mandibular first molar of *Bmp9*-KO mice is shorter, the apical foramen is wider, and the dentin is thinner. We further confirm that *Bmp9* regulates the differentiation and secretion of odontoblasts in the root region. Ablation of *Bmp9* reduces cell proliferation and increases intercellular junctions in HERS, which further influences the development of root dentin and the closure of the apical foramen. Our results show that BMP9 signaling can regulate odontoblasts and HERS and thereby further regulate root development and providing insights for future root regeneration strategies.

## Materials and methods

### Generation of *Bmp9*-KO mice

Hybrid varieties of *Bmp9*^+/−^ were sourced from Cyagen Model Biological Center Co., Ltd., located in Suzhou, China. Wild-type (WT; *Bmp9*^+/+^) and *Bmp9* knockout (*Bmp9*^*−*/−^) mice utilized in the experimental procedures were derived from breeding *Bmp9*^+/−^ individuals. Ethical clearance for all animal experiments was granted by the Ethics Committee of the Affiliated Hospital of Stomatology at Chongqing Medical University, under reference CQHS-REC-2023 (LSNo.69).

### Western blotting analysis

Samples were lysed with a radioimmunoprecipitation assay (RIPA) buffer (Beyotime, China). The protein concentrations were measured using the BCA Protein Assay Kit (Beyotime). Protein lysates (25 μg per lane) were separated by SDS-PAGE and then transferred onto nitrocellulose membranes (Millipore, USA). The membranes were incubated sequentially with primary antibodies specific to BMP9 (1:500, Thermo Fisher), followed by secondary antibodies (1:2000, ZSGB, China). Visualization was performed using a chemiluminescence detection kit from Beyotime, China.

### Quantitative PCR analysis

RNA extraction was performed using the Universal RNA Extraction Kit (Takara, Japan), and complementary DNA (cDNA) synthesis was conducted with a reverse transcription kit (Takara). The following PCR primers were employed: for mouse *Bmp9*, CCGCAGTACATGATTGACCTG and CTGTGGCAGTTATGGAGATGG; for mouse cytokeratin-14 (*Ck14*), GTCAATGTGGAGATGGACGC and AATCTCACTCTTGCCGCTCT; for mouse *E-cadherin*, CAAAGTGACGCTGAAGTCCA and TACACGCTGGGAAACATGAG; for mouse *Vimentin*, AGATGCGTGAGATGGAAGA and TCCAGCAGCTTCCTGTAGGT; for mouse *Ki67*, CAGTACTCGGAATGCAGCAA and CAGTCTTCAGGGGCTCTGTC; for mouse zonula occludens-1 (*Zo-1*), TATCCAAACCAGACCCACCC and GGCTTTGGTGTGAATCGGTT; and for mouse *Actb*, CTGGCTCCTAGCACCATGA and CCTGCTTGCTGATCCACAT. The relative gene expression levels were determined using the 2^−ΔΔCt^ method, with normalisation to *Actb* for standardisation.

### Hematoxylin-eosin and immunohistochemistry staining

Mandibular samples were prepared through fixation, demineralization, paraffin embedding, sectioning, and staining with hematoxylin and eosin (Solarbio, China). The sections underwent antigen retrieval, permeabilization, and blocking following standard protocols. Primary antibodies were applied at 4 °C overnight, including BMP9 (1:100, Thermo Fisher, PA5-11931), DSPP (1:200, Abcam, ab216892), and Nestin (1:100, Bioss, bs-0008R). The following day, sections were treated with a secondary antibody kit (ZSGB, China, PV-9001) and visualized with diaminobenzidine (DAB, Sigma–Aldrich).

### Histology and stereomicroscopy

The mandibular molars were imaged using a high-resolution inverted microscope (Olympus DSX500i).

### Micro-computed tomography (micro-CT) analysis

The mandible was imaged using a micro-CT scanner (Viva CT 40, Scanco Medical, Bassersdorf, Switzerland) operating at 70 kVp and 114 μA, with a resolution of 15 μm voxel size. Digital images obtained were processed and analyzed using Mimics 17.0 software (Materialise NV, Leuven, Belgium). Three-dimensional reconstructions and comparative re-sectioning were conducted on both *Bmp9*-KO and WT mice to evaluate structural differences.^25^

### Sirius red staining

The paraffin sections were stained with Sirius red dye for 8–10 min, after which they were sealed with neutral gum gel.^26^

### Immunofluorescent staining

Sections were permeabilized using 4% paraformaldehyde for 15 min, followed by treatment with 0.2% Triton-X100 for an additional 15 min. After a 1-h blocking step with 10% goat serum, the samples were incubated at 4 °C overnight with primary antibodies diluted in phosphate-buffered saline solution as per the following specifications: BMP9 (1:200, Thermo Fisher, PA5-11931), CK14 (1:100, Abcam, ab7800), Ki67 (1:200, Abcam, ab15580), and ZO-1 (1:200, Abcam, ab221547). After incubation, sections were rinsed three times with phosphate-buffered saline solution and then incubated with fluorescence-conjugated secondary antibodies for 1 h. The secondary antibodies used were goat anti-rabbit DyLight488 (1:500, Abbkine, A23220) and goat anti-mouse DyLight594 (1:500, Abbkine, A23310). Finally, sections were counterstained with DAPI (Beyotime, China) for nuclear visualization. Images were captured using a confocal microscope (LSCM, Leica).

### Cell culture and recombinant adenoviruses

Primary HERS cells were isolated from the neck tissue located beneath the first molar crown of C57BL/6 mice on postnatal day 7. The tissue was enzymatically digested at 37 °C using a solution containing 3 mg/mL collagenase type I and 4 mg/mL dispase for 1 h. Mesenchymal stem cells were extracted from the tissue of the first molar crown and digested with 1 mg/mL collagenase type I at 37 °C for 30 min. The cells were cultured in alpha minimum essential medium (Sigma–Aldrich, St. Louis, Missouri, USA) supplemented with 10% fetal bovine serum (GIBCO, California, USA), 1% penicillin (100 U/mL), and streptomycin (100 μg/mL, Sigma–Aldrich), maintained at 37 °C in a 5% CO_2_ environment. The medium was changed every two days, and mesenchymal stem cells were separated by trypsin gradient digestion.

For adenovirus amplification, the 293 pTP cell line was utilized. These cells were cultured in complete Dulbecco's modified Eagle's medium (Sigma–Aldrich, St. Louis, Missouri, USA) at 37 °C in a 5% CO_2_ atmosphere, following standard protocols. Recombinant adenoviruses overexpressing BMP9, green fluorescent protein (GFP), or siBMP9 (BMP9 silencing) were generated according to previously established methods.^27^ The Ad-GFP virus served as a control.^28^^,^^29^ Transduction efficiency was enhanced using 5 μg/mL polybrene (Sigma–Aldrich, St. Louis, Missouri, USA).^30^

### Proliferation assay

HERS cells were transfected with Ad-GFP and Ad-BMP9 and subsequently seeded into 96-well plates at a density of 1 × 10^3^ cells per well. Cell proliferation was evaluated using the Cell Counting Kit-8 (CCK-8) assay (Bioss, China) on days 1, 3, 5, and 7. The absorbance of the reaction products was measured spectrophotometrically at 450 nm to quantify cell viability.

### Adenovirus injection

Newborn mice were administered Ad-siBMP9 injections on the day of birth using a microsyringe (Boli Pigeon Industry & Trade, China).^31^ Guided by an SZX 10 microscope (Olympus), 10 μL of either Ad-siBMP9 or control Ad-GFP solution was precisely injected into the mesial root of the mandibular first molar. At 14 and 28 days after injection, the mandibular molar roots were subjected to histological examination, micro-CT scanning, and subsequent 3D reconstruction analysis to assess structural changes.

### RNA sequencing analysis

Dental papillae tissues were collected separately from *Bmp9*-KO mice and WT mice for RNA sequencing analysis. Total RNA was extracted using the RNA Extraction Kit (Invitrogen). RNA quality control included absorbance ratios of A260/A280 = 1.8–2.2, RNA integrity number (RIN) > 7, and 29S:18S RNA ribosomal RNA ratios > 1.5 for downstream processing. Strand-specific cDNA libraries were prepared with the VAHTS™ Universal V6 RNA-Seq Library Prep Kit, and sequencing was performed on the Illumina Nova-Seq 6000 Platform in PE150 (paired-end 150-bp reads) mode. Then, the batch effect was removed using the ComBat method in the R package “sva”. Differential expression analysis was conducted using DESeq2 (v1.38.3), with thresholds defined as |log_2_ (fold-change)| > 0.5 and *P*-values < 0.05. The transcriptome sequencing was executed by Majorbio Bio-pharm Technology Co., Ltd., Shanghai, China.

### Statistical analysis

The data were presented as mean ± standard deviation. Statistical analyses were conducted using GraphPad Prism 9. Differences between two groups were assessed using Student's *t*-test. For comparisons among multiple groups, one-way analysis of variance (ANOVA) was applied, followed by Tukey's multiple comparison test for evaluating mean differences.

## Results

### *Bmp9* plays a critical role in mouse tooth root morphogenesis

**Figure 1:**
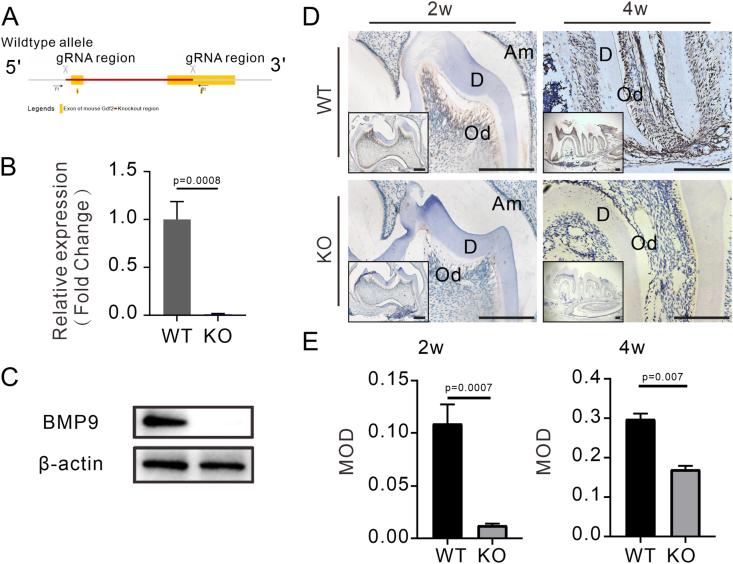
*Bmp9* knockout (KO) mouse model was successfully established. **(A)** Construction of the *Bmp9*-KO mice. **(B)** Relative expression of *Bmp9* in the liver of 8-week-old wild-type (WT) littermates and *Bmp9*-KO mice by quantitative PCR *in vitro* (*n* ≥ 3). **(C)** Western blots of BMP9 and β-actin in the liver of 8-week-old WT littermates and *Bmp9*-KO mice (*n* = 6). **(D)** Immunohistochemical staining of BMP9 on sections of mandibular first molars from WT littermates and *Bmp9*-KO mice at 2 weeks and 4 weeks old (*n* = 7). The boxes shown are insets at a lower magnification. Am, ameloblasts; Od, odontoblasts; D, dentin. **(E)** Quantitative analysis of BMP9 expression. Scale bars = 200 μm.

To elucidate the role of BMP9 signaling during molar root development, we generated *Bmp9-*KO mice (Fig. 1A). To validate the successful establishment of the *Bmp9*-KO mouse model, we quantified *Bmp9* mRNA expression in the liver tissue of *Bmp9*-KO mice and WT littermates at 8 weeks by quantitative reverse transcription PCR analysis. The results demonstrated a significant reduction in *Bmp9* expression in *Bmp9*-KO mice relative to WT controls, which was further corroborated by western blotting analysis (Fig. 1B, C). During tooth root development, BMP9 expression was broadly detected in the odontoblasts, ameloblasts, and a subset of dental pulp cells in WT mice at 2 weeks and 4 weeks (Fig. 1D). In contrast, BMP9 expression was markedly diminished in *Bmp9*-KO mice, as confirmed by both immunohistochemical staining and quantitative analysis (Fig. 1D, E). These findings suggest a critical role for BMP9 in molar root development, with its loss leading to substantial changes in cellular expression patterns.

**Figure 2:**
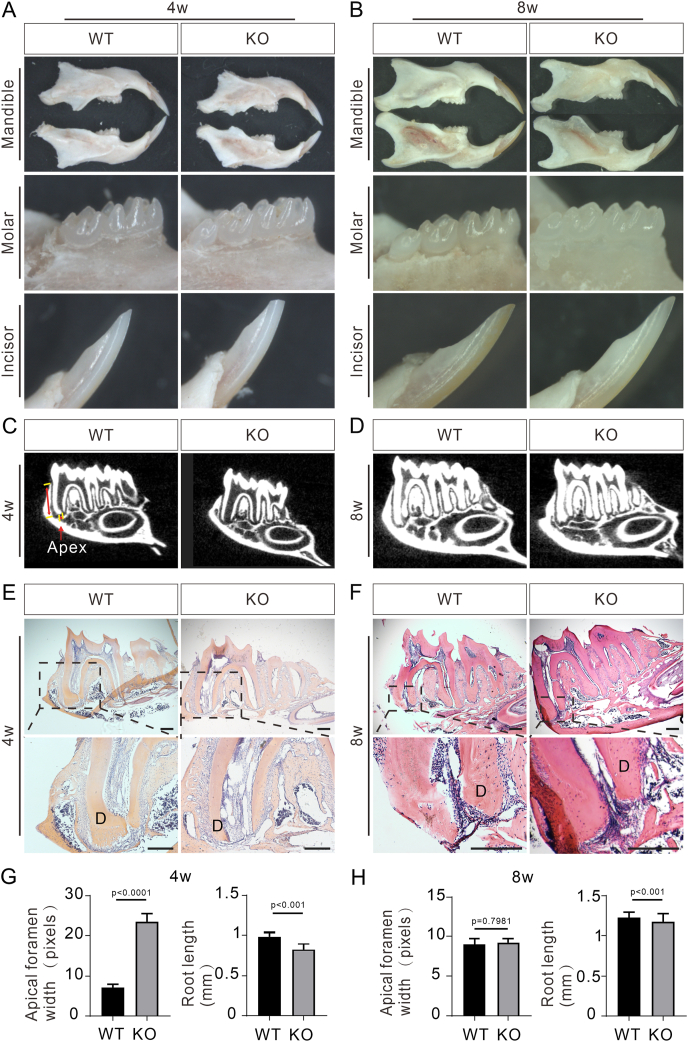
*Bmp9* plays a critical role in mouse molar root morphogenesis. **(A, B)** Gross morphology of the incisors and mandibular molars of wild-type (WT) littermates and *Bmp9* knockout (KO) mice at 4 weeks (A) and 8 weeks (B) (*n* = 7). **(C, D)** micro-CT images of mandibular first molars of WT littermates and *Bmp9*-KO mice at 4 weeks (C) and 8 weeks (D) (*n* = 6). Double-headed arrows indicate root length and apical foramen width. **(E, F)** Hematoxylin-eosin staining of mandibular first molars of WT littermates and *Bmp9*-KO mice at 4 weeks (E) and 8 weeks (F) (*n* = 7). The dashed-line boxes are enlarged. D, dentin. **(G, H)** Quantification analysis of the root length and width of the apical foramen in the mandibular first molars of WT littermates and *Bmp9*-KO mice at 4 weeks (G) and 8 weeks (H). Scale bars = 200 μm.

To determine the functional significance of *Bmp9*-mediated signaling in molar root development, we conducted a comparative analysis of the gross tooth morphology in *Bmp9*-KO mice and their WT littermates at 4 and 8 weeks of age (Fig. 2A, B). At 4 weeks, we observed that the incisor tips of *Bmp9*-KO mice were prone to fracture, indicating compromised dentin mineralization and reduced hardness in the absence of *Bmp9* (Fig. 2A, B). By 8 weeks, WT mice exhibited yellow incisors, indicative of proper mineralization, while *Bmp9*-KO mice displayed white incisors with significantly reduced mineralization (Fig. 2A, B). At 4 weeks, the development of the dental crown was nearly complete. Both *Bmp9*-KO and WT mice had a comparable number of tooth cusps on their molars, with no noticeable signs of wear (Fig. 2A, B). These experimental results indicate that ablation of *Bmp9* has minimal impact on the early development of mouse molar crowns, but exerts a significant effect on the mineralization degree of incisor dentin. Thus, *Bmp9* appears to play a crucial regulatory role in the mineralization of dentin and the development of tooth roots during early odontogenesis.

To further investigate the role of *Bmp9* in the early development of mouse molar roots, micro-CT analysis was performed on the teeth of *Bmp9*-KO mice and their WT littermates at 4 weeks and 8 weeks of age (Fig. 2C, D). Quantitative analysis of the image data revealed that the mandibular first molars of 4-week-old *Bmp9*-KO mice exhibited significantly shorter roots and a wider apical foramen compared with WT mice (Fig. 2G). Additionally, *Bmp9*-KO mice showed a marked widening of the root canal and a notable thinning of the dentin (Fig. 2C). At 8 weeks, the apical foramen of the mandibular first molar in *Bmp9*-KO mice and WT mice approached closure (Fig. 2D); however, root length remained significantly reduced in the *Bmp9*-KO mice (Fig. 2H). At 4 weeks and 8 weeks, histologic analysis confirmed that ablation of *Bmp9* led to shortened molar roots, while the root number remained normal in the mandibular first molars (Fig. 2E, F). Therefore, the above results indicate that *Bmp9* plays an important regulatory role in the development of mouse molar teeth, particularly influencing root elongation and dentin mineralization during early tooth development.

### Loss of *Bmp9* hinders differentiation and secretion of odontoblasts

**Figure 3:**
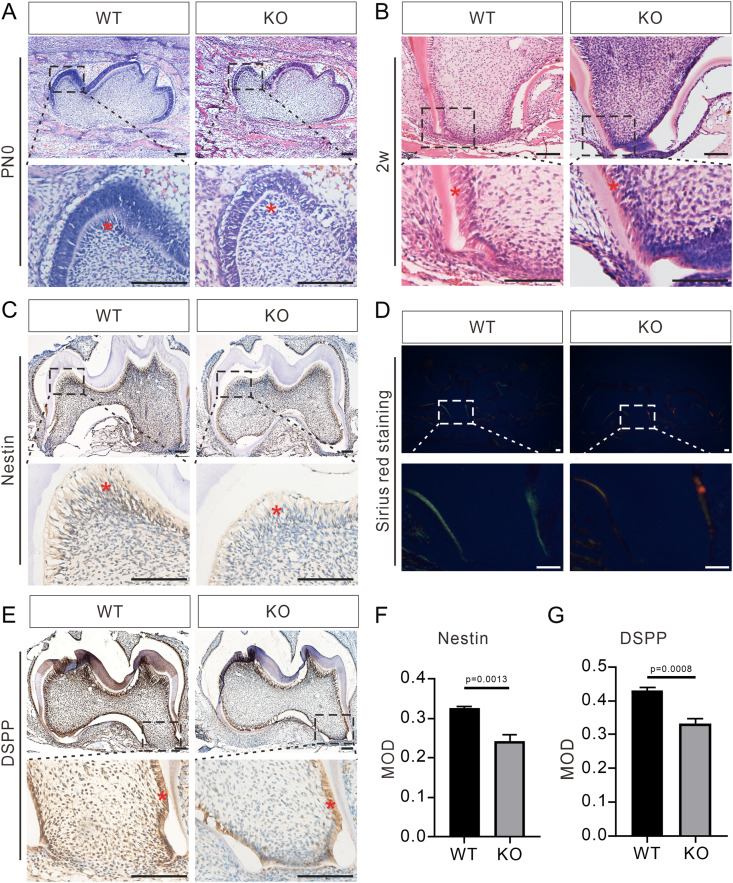
Loss of *Bmp9* hinders the differentiation and secretion of odontoblasts. **(A, B)** Hematoxylin-eosin staining of mandibular first molars of wild-type (WT) littermates and *Bmp9* knockout (KO) mice on postnatal day 0 (PN0) (A) and at 2 weeks (B) (*n* = 7). The dashed-line boxes are enlarged. The asterisks indicate the odontoblasts. **(C)** Immunohistochemical staining of nestin in the mandibular first molars of WT littermates and *Bmp9*-KO mice at 2 weeks of age (*n* = 6). The dashed-line boxes are enlarged. The asterisks indicate the odontoblasts. **(D)** Sirius red staining of mandibular first molars of WT littermates and *Bmp9*-KO mice at 2 weeks of age (*n* = 6). The dashed-line boxes are enlarged. **(E)** Immunohistochemical staining of DSPP in the mandibular first molars of WT littermates and *Bmp9*-KO mice at 2 weeks of age (*n* = 6). The dashed-line boxes are enlarged. The asterisks indicate the odontoblasts. **(F, G)** Quantitative analysis of nestin and DSPP expression. Scale bars = 100 μm.

Compared with WT mice, *Bmp9*-KO mice exhibited significant developmental abnormalities, including poor dentin mineralization, shortened tooth roots, and thinner dentin. To further investigate the regulatory effect of *Bmp9* in odontoblast differentiation and secretions, we focused on the mandibular first molars as a primary research model. In *Bmp9*-KO mice, no mesenchymal cells differentiating into odontoblasts were observed at the tips of the mandibular first molars on postnatal day 0, indicating impaired odontoblast differentiation compared with WT controls (Fig. 3A). Further comparison revealed that the predentin layer of *Bmp9*-KO mice was wider than that of control mice at 2 weeks, indicating abnormal matrix secretion and a reduced mineralizing capacity of odontoblasts in *Bmp9*-KO mice, contributing to reduced dentin mineralization (Fig. 3B). Additionally, odontoblasts in *Bmp9*-KO mice exhibited a looser alignment compared with the densely packed, columnar arrangement seen in WT molars, suggesting abnormalities in intercellular junctions (Fig. 3B). The amount of matrix secreted by the cells was normal or slightly reduced, indicating that the widening of the predentin in *Bmp9*-KO mice may result from impaired mineralization due to aberrant protein secretion.^32^ To clarify the underlying cause of the diminished odontoblast differentiation in *Bmp9*-KO mice, we detected the expression of nestin, a well-established marker of odontoblast polarization.^33^ At 2 weeks, only a few nestin-positive odontoblasts were detected in the mandibular first molars of *Bmp9*-KO mice, indicating a disruption in odontoblast polarization (Fig. 3C). Quantitative analysis confirmed significant differences in nestin expression between *Bmp9*-KO and WT mice (Fig. 3F). These findings demonstrate that the ablation of *Bmp9* inhibits odontoblast differentiation and interferes with the normal development of the mandibular first molars, particularly in their ability to form and mineralize dentin.

Collagen type I, the predominant component of the dentin matrix, plays a crucial role in regulating the rate and outcome of dentin deposition and serves as an indicator of odontoblast secretion function.^34^ To assess the impact of *Bmp9* ablation on collagen type I expression, we performed Sirius red staining on the mandibular first molars of 2-week-old *Bmp9*-KO mice and WT controls. Our analysis revealed a marked increase in collagen type I deposition in *Bmp9*-KO mice compared with controls (Fig. 3D). Given the absence of significant differences in dentin morphology and thickness at 8 weeks, we speculate that the slower dentin mineralization in *Bmp9*-KO mice may lead to an accumulation of unmineralized collagen type I. Additionally, we investigated the expression of DSPP, a marker of terminally differentiated odontoblasts, in *Bmp9*-KO mice and control mice at 2 weeks. Our findings demonstrated a significant reduction in DSPP expression in the odontoblasts of the mandibular first molars in *Bmp9*-KO mice (Fig. 3E). Quantitative analysis further confirmed these differences (Fig. 3G). These results indicate that the loss of *Bmp9* severely impairs the secretory function of odontoblasts, leading to compromised dentin mineralization. Consequently, *Bmp9* deficiency results in aberrant root development in the molars of *Bmp9*-KO mice, likely due to diminished dentin mineralization capacity and altered odontoblast function.

### Ablation of *Bmp9* reduces cell proliferation and enhances intercellular junctions in HERS

**Figure 4:**
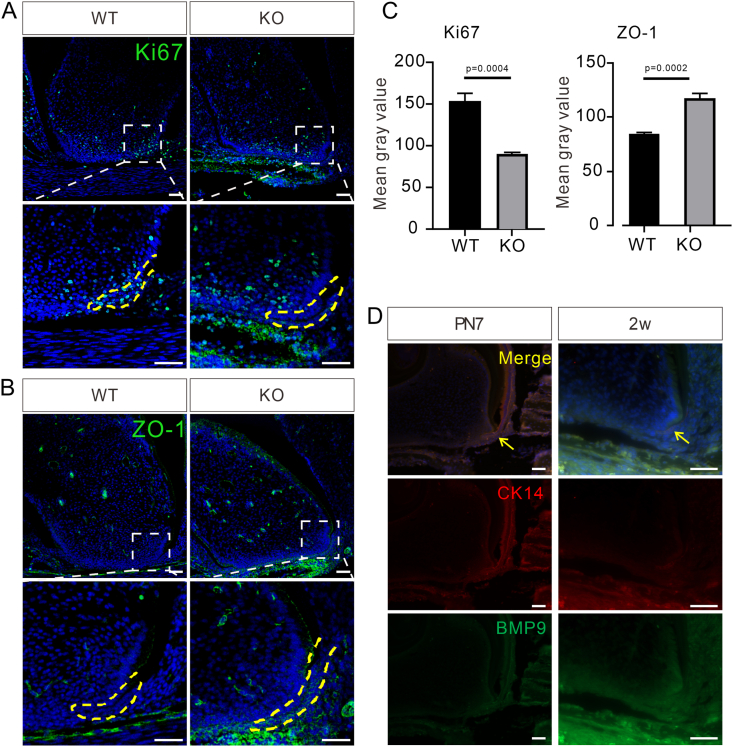
Ablation of *Bmp9* reduces cell proliferation and enhances intercellular junctions in HERS. **(A, B)** Immunofluorescent staining of Ki67 (A) and ZO-1 (B) in the mandibular first molars of wild-type (WT) littermates and *Bmp9* knockout (KO) mice at 2 weeks of age (*n* = 6). The dashed-line boxes are enlarged. The yellow line represents HERS. **(C)** Quantitative analysis of Ki67 and ZO-1 expression. **(D)** Immunofluorescent staining of CK14 and BMP9 in the mandibular first molars of WT littermates on postnatal day 7 (PN7) and at 2 weeks (*n* = 6). The arrows indicate HERS. Scale bars = 50 μm.

The interaction between HERS and mesenchymal cells is a critical factor initiating root dentin formation. Specifically, the inner layer cells of the HERS trigger the differentiation of odontoblasts, which subsequently form root dentin. To investigate the impact of *Bmp9* deletion on cell proliferation during root development, we assessed the proliferative activity in molar teeth. Immunofluorescence staining revealed a significant reduction in HERS proliferation in 2-week-old *Bmp9*-KO mice compared with WT controls (Fig. 4A). This diminished proliferation likely interferes with the normal elongation and bending of HERS, a process essential for proper root formation. In conjunction with these findings, the previous results indicated that the odontoblasts in *Bmp9-*KO molars exhibited a looser alignment. To explore the potential mechanisms underlying these alterations, we examined the expression of ZO-1, a marker for tight junctions. Our analysis revealed that the intercellular junctions were notably enhanced in the molar odontoblasts of 2-week-old *Bmp9-*KO mice compared with controls (Fig. 4B), with quantitative analysis confirming significant differences (Fig. 4C). Reduced proliferation may regulate the process of HERS elongation and bending, directing the formation of the root apical foramen, resulting in shorter roots, thinner dentin, and an unclosed apical foramen in *Bmp9*-KO mandibular first molars.

To further investigate the hypothesis that *Bmp9* deficiency leads to reduced proliferation and enhanced intercellular junctions in HERS, ultimately influencing normal tooth root development, we examined the expression of BMP9 in the HERS of WT mice on postnatal day 7 and at 2 weeks. The results revealed an overlap in the expression of BMP9 and CK14 in the mandibular first molar root, indicating that *Bmp9* may play a role in guiding root development through the interaction between HERS and mesenchymal cells (Fig. 4D).

### *Bmp9* regulates the proliferation and intercellular junctions of primary HERS cells

**Figure 5:**
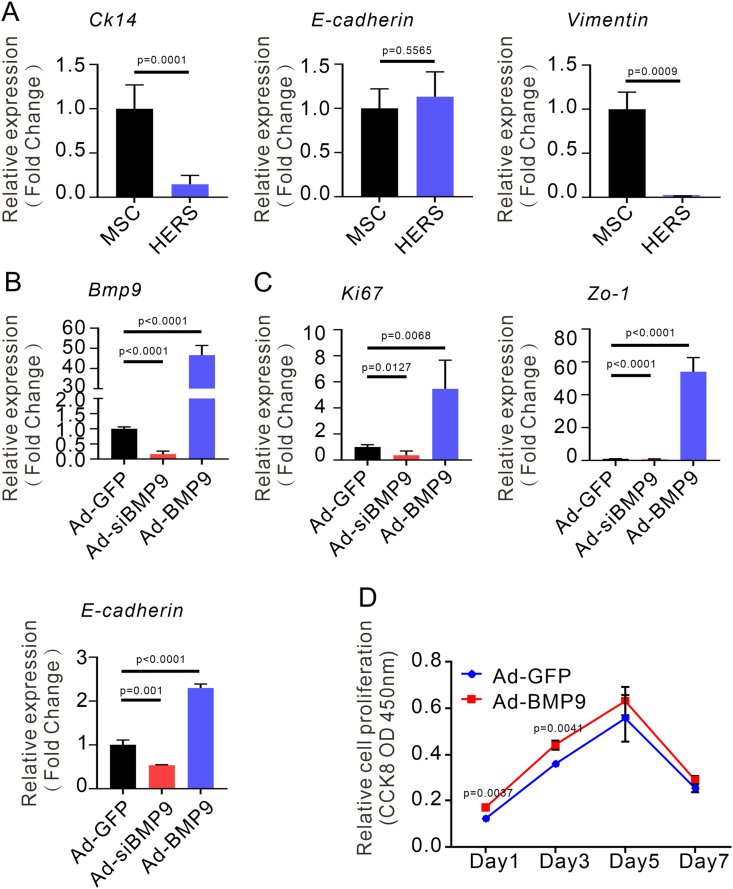
*Bmp9* regulates the proliferation and intercellular junctions of primary HERS cells. **(A)** Relative expression of *Ck14*, *E-cadherin*, and *Vimentin* in the mesenchymal stem cells (MSC) and HERS cells by quantitative PCR (*n* = 3). **(B, C)** After silencing/overexpression of *Bmp9*, relative expression of *Bmp9* (B), *Ki67* (C), *E-cadherin* (C), and *Zo-1* (C) in primary HERS cells by quantitative PCR (*n* = 3). **(D)** The proliferation rate of HERS infected with Ad-GFP and Ad-BMP9 measured by CCK-8 assay (*n* = 3).

**Figure S1:**
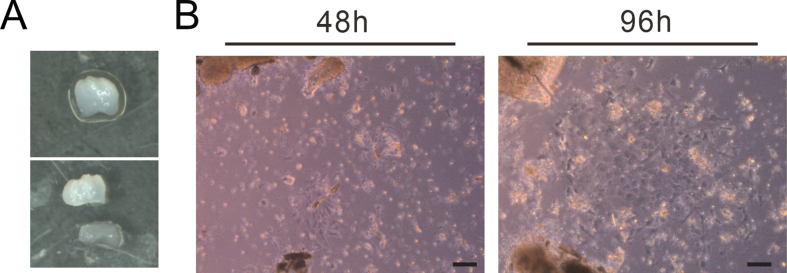
Primary HERS cells. **(A)** Crown and neck tissues of mouse molars were obtained. **(B)** Morphological observations of mouse primary HERS cells. Scale bar = 200 μm.

Based on the method of primary HERS extraction in the early stage, we extracted primary HERS cells (Fig. S1). The extracted primary HERS cells exhibited positive *Ck14* expression, high levels of *E-cadherin*, and minimal *Vimentin* expression, consistent with previously reported characteristics of HERS cells (Fig. 5A). This demonstrates the successful isolation of primary HERS cells, providing a robust *in vitro* model for studying the molecular mechanisms by which *Bmp9* regulates root development via its effects on HERS proliferation and intercellular junctions.

To verify the role of *Bmp9* in tooth root development, we overexpressed it in HERS cells by transfecting Ad-BMP9 and confirmed the efficient overexpression of *Bmp9* by quantitative reverse transcription PCR analysis (Fig. 5B). Simultaneously, Ad-siBMP9 was transfected, and the silencing of *Bmp9* expression in HERS cells was confirmed by quantitative reverse transcription PCR analysis (Fig. 5B). Upon *Bmp9* overexpression, we observed that the expression level of proliferation biomarker *Ki67* was robustly elevated, paralleled by a marked up-regulation of *Zo-1* and *E-cadherin* expression (Fig. 5C). In contrast, when *Bmp9* was down-regulated using Ad-siBMP9 in HERS cells, the proliferation and the intercellular junction of primary HERS cells was notably reduced (Fig. 5C). To further assess the impact of *Bmp9* on HERS cell proliferation, we employed the CCK-8 assay. The results showed that *Bmp9* overexpression significantly promoted the proliferation of mouse primary HERS cells (Fig. 5D). The results demonstrate that BMP9 signaling can regulate the proliferation of mouse primary HERS cells, which, in turn, influences the development of mouse molar roots. Interestingly, while ZO-1 expression was significantly elevated in the HERS of 2-week-old *Bmp9*-KO mouse molars, the expression of *Zo-1* in primary HERS cells was relatively low upon extraction. This discrepancy may be attributed to the *in vitro* growth environment, which could influence *Zo-1* expression in cultured cells. Collectively, these results highlight that BMP9 signaling modulates both HERS cell proliferation and intercellular junctions, thereby contributing to the proper development of tooth roots.

### Silencing *Bmp9* expression inhibits root development *in vivo*

**Figure 6:**
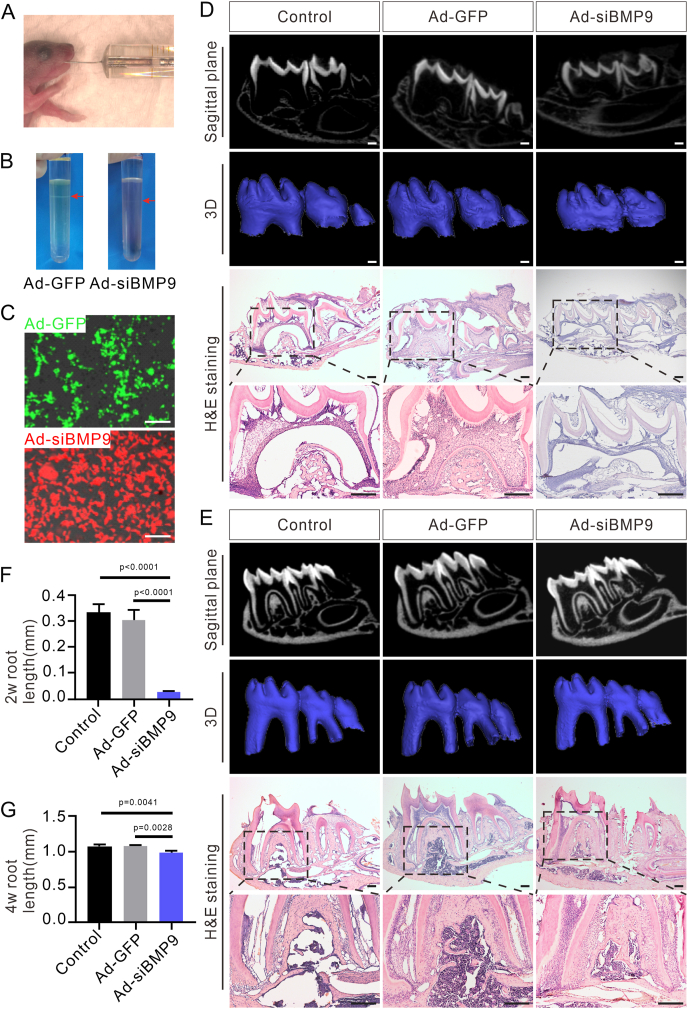
Silencing *Bmp9* expression inhibits root development *in vivo*. **(A)** Ad-GFP/Ad-siBMP9 is injected directly into the mandibular first molars of wild-type mouse littermates (*n* = 6). **(B)** Concentrated adenovirus by ultracentrifugation. **(C)** Adenovirus expression is detected by cell fluorescence. **(D, E)** micro-CT images, 3D reconstruction, and hematoxylin-eosin staining of control, Ad-GFP, and Ad-siBMP9 mandibular molars after 2 weeks (D) and 4 weeks (E) of adenovirus injection since postnatal day 0 (*n* = 6). The dashed-line boxes are enlarged. **(F, G)** Quantitative analysis of root length of mandibular first molars in control, Ad-GFP, and Ad-siBMP9 groups at 2 weeks (F) and 4 weeks (G). Scale bars = 200 μm.

Preliminary experiments have demonstrated that *Bmp9* ablation inhibits the differentiation and secretion functions of odontoblasts. To further validate the functional role of *Bmp9* in tooth root development, we employed direct injections of Ad-GFP or Ad-siBMP9 into the mandibular first molars of WT mice, which naturally express high level of *Bmp9* (Fig. 6A). After amplification, concentration, and purification, the adenovirus was administered directly into the mesial region of the first molars (Fig. 6B, C). Mandibular tissues were harvested at 2 and 4 weeks after injection to assess differences in root development. micro-CT images and 3D reconstruction revealed that the root length of the mandibular first molar in the Ad-siBMP9 group was notably shorter compared with that in the Ad-GFP group at 2 weeks (Fig. 6D). No significant difference was observed between the Ad-GFP group and the control group (Fig. 6F). At 4 weeks, root length in the Ad-siBMP9 group remained significantly shorter than in both the Ad-GFP group and the control group (Fig. 6E). Quantitative analysis, measuring the root length from the amelocemental junction to the apical foramen, confirmed that the mandibular first molars roots in the Ad-siBMP9 group were significantly shorter than those in the Ad-GFP group and the control group at both 2 and 4 weeks (Fig. 6F, G). Histological analysis supported the results of micro-CT and 3D reconstruction, further confirming that *Bmp9* silencing led to shorter roots in the mandibular first molars compared with normal mice (Fig. 6D, E). These findings indicate that silencing *Bmp9* expression results in developmental abnormalities in mouse molars, particularly characterized by shortened roots, confirming the critical role of *Bmp9* in tooth root development.

**Figure 7:**
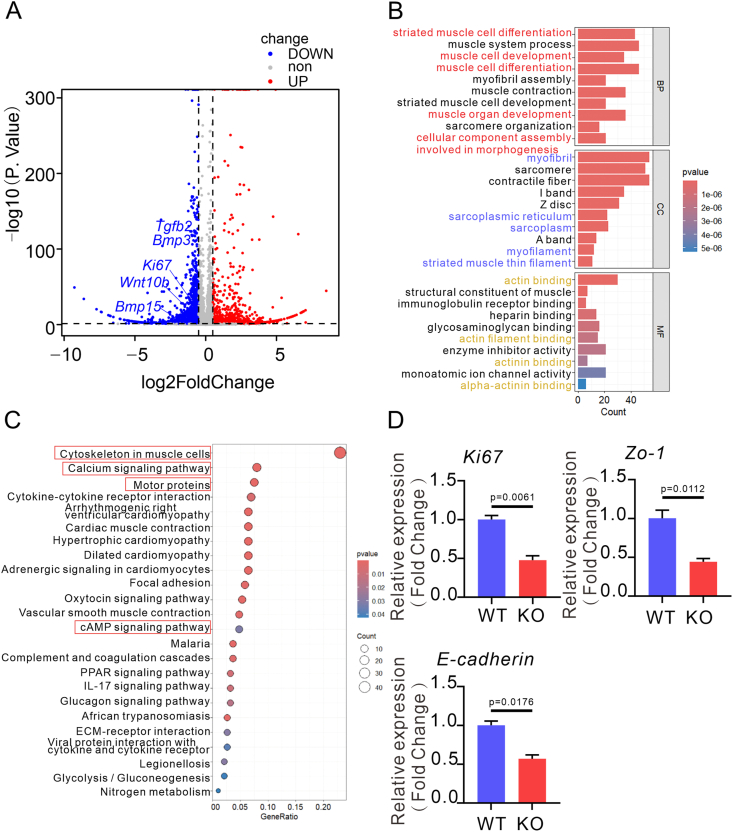
Potential mechanism and validation of knocking out *Bmp9* to inhibit cell proliferation and intercellular junctions. **(A)** The volcano diagram portraying the gene expression profile between *Bmp9* knockout (KO) mice and wild-type (WT) controls in the root apical papilla. Up-regulated genes were marked in red and down-regulated genes were marked in blue. **(B)** Top 10 pathways from the GO enrichment analysis. The top 10 items of biological processes (BP), the top 10 items of molecular functions (MF), and the top 10 items of cellular components (CC) were shown. **(C)** The top 25 items of KEGG pathway. **(D)** The relative mRNA levels of *Ki67*, *Zo-1*, and *E-cadherin* (*n* = 3).

To further elucidate the molecular mechanisms underlying *Bmp9*-mediated root morphogenesis, we employed RNA sequencing to investigate the molecular consequences of *Bmp9* ablation during odontogenesis. Comparative transcriptomic profiling revealed 2289 down-regulated and 1066 up-regulated differentially expressed genes (DEGs) in the *Bmp9*-KO cohort relative to WT controls (Fig. 7A). Volcanic diagram analysis demonstrated significant down-regulation of TGF-β superfamily members (*Tgf-β2*, *Bmp3*, *Bmp15*) and Wnt/β-catenin pathway ligand *Wnt10b* in root canine papilla tissues of *Bmp9*-KO mice, indicating that *Bmp9* deficiency disrupts signaling synergy between BMP/TGF-β pathways and impairs Wnt-mediated epithelial-mesenchymal crosstalk (Fig. 7A). Additionally, *Dkk1*, *Wnt3a*, *Wnt7a*, and *Smad3* were up-regulated, alongside down-regulation of *Dkk2*, *Dmp1*, *Dspp*, *Wnt10b*, and *Dmp1* in *Bmp9*-KO mice. To mechanistically interrogate how *Bmp9* ablation perturbs root morphogenesis, we performed functional annotation of these DEGs using Gene Ontology (GO) and Kyoto Encyclopedia of Genes and Genomes (KEGG) pathway enrichment analyses, thereby identifying critical biological processes disrupted by *Bmp9* deficiency. GO enrichment analysis revealed that DEGs were significantly enriched in biological processes related to cell differentiation, organ development, and morphogenesis (Fig. 7B). High-confidence enrichment was observed for biological processes including muscle cell differentiation, muscle organ development, and cellular component assembly involved in morphogenesis (red marks), suggesting that *Bmp9* may regulate the expression dynamics of cytoskeletal proteins (*e.g.*, actin and myosin) to mediate cellular polarity establishment and migration remodeling, thereby indirectly affecting intercellular junction integrity. In the cellular component category, DEGs were specifically enriched in myofibril-associated ultrastructures, particularly the sarcoplasmic reticulum calcium reservoir regulatory system (blue marks). Molecular function analysis further demonstrated significant enrichment of DEGs in actin-binding domains (yellow marks), indicating that *Bmp9* may coordinate the spatiotemporal coupling of proliferative activity and junctional remodeling by modulating the dynamic polymerization/depolymerization equilibrium of actin filaments, thereby influencing the cytoskeleton. Consistently, KEGG pathway enrichment analysis demonstrated that DEGs were significantly enriched in core pathways related to cell proliferation and intercellular junctions, including cytoskeleton in muscle cells, calcium signaling pathway, motor proteins, and cAMP signaling pathway (red marks) (Fig. 7C). Mechanistically, *Bmp9* maintains HERS cell proliferation-junction homeostasis and drives root morphogenesis by integrating a multidimensional regulatory network encompassing cytoskeletal tension (mechanical signaling), calcium flux (chemical signaling), and motor protein trafficking (spatial signaling). Quantitative reverse transcription PCR validation showed a coordinated down-regulation of proliferation marker *Ki67*, tight junction protein *Zo-1*, and epithelial polarity regulator *E-cadherin* in *Bmp9*-KO apical incisor tissues, implicating that *Bmp9* deletion induces partial loss of proliferative-differentiation balance and disrupts intercellular junction integrity (Fig. 7D). This phenotype is highly consistent with the predicted defects in cytoskeleton recombination and abnormal calcium signaling by transcriptome analysis. The results elucidate the molecular mechanism by which *Bmp9* maintains stable tooth root development by regulating the multi-pathway interaction network (TGF-β/Wnt/cell connections), providing a new direction for targeted regulation of tooth root regeneration based on *Bmp9*.

## Discussion

Previous studies have highlighted the significance of various signaling pathways, such as BMP, TGF-β, WNT, FGF, and SHH, in orchestrating root formation. Specifically, up-regulation of *Bmp6* expression serves as the basis for a significant increase in tooth numbers,^21^ knocking out *Bmp2* and *Bmp4* in mouse epithelium results in shortened tooth roots.^22^ Interestingly, after *Bmp9* knockout, members of the TGF-β superfamily (*Tgf-β2*, *Bmp3*, *Bmp15*) and Wnt/β-catenin pathway ligand *Wnt10b* were significantly down-regulated in the root canine papilla tissue of mice, indicating that *Bmp9* deficiency disrupts the signal synergy between the BMP/TGF-β pathway and impairs Wnt-mediated epithelial-mesenchymal crosstalk. Despite extensive research on these pathways, the specific role of BMP9 signaling in root development has remained largely uncharted. BMP9 is known to influence cellular processes, including proliferation and differentiation.^35^, ^36^, ^37^, ^38^, ^39^ In this study, we demonstrate that *Bmp9* is instrumental in regulating both cell proliferation and intercellular junctions within the HERS of mouse molars. This regulatory role of BMP9 signaling contributes to essential aspects of root morphogenesis, influencing root length, apical foramen width, and dentin thickness during tooth development.

Interestingly, the analysis of RNA sequencing results of apical incisor tissues in *Bmp9*-KO mice and WT littermates identified up-regulation of *Dkk1*, *Wnt3a*, *Wnt7a*, and *Smad3*, alongside down-regulation of *Dkk2*, *Dspp*, *Wnt10b*, and *Dmp1* in *Bmp9*-KO mice. These results suggest that *Bmp9* loss leads to reduced expression of *Dspp* and *Dmp1*, implying diminished odontoblast differentiation and mineralization capacity in these tissues, which aligns with the findings of our current study. Additionally, actin levels were found to be increased. Actin filaments typically integrate into the cytoplasm of adjacent enamel cells via cell junction complexes, forming a terminal web critical for enamel formation. Consequently, the precise role of increased actin in regulating odontoblast differentiation and mineralization warrants further investigation to clarify its impact on tooth development. Upon contact with the basement membrane on the inner side of the HERS, dental papilla cells differentiate into odontoblasts. During dentin formation, dental follicle cells infiltrate the disrupted HERS, make contact with the newly formed dentin, and differentiate into cementoblasts, which initiate cementum deposition on root dentin.^40^ *Wnt10a* knockout, both in the entire tissue and specifically in dental epithelium, results in the absence or apical displacement of root furcation in mouse molars, creating a taurodontism-like phenotype. Studies further reveal that *Wnt10a*-KO mice exhibit significantly reduced proliferation of inner enamel epithelium cells, leading to abnormal root furcation, which aligns with our study's findings.^41^ Additionally, inducible tissue-specific *β-catenin* conditional knockout mice (*Ctnnb1*^iΔshh^) have shown that *β-catenin* inactivation in HERS disrupts root elongation due to premature HERS degradation. This phenotype is associated with decreased cell–cell adhesion, reduced junctional protein expression, and increased epithelial-to-mesenchymal transition in HERS cells upon *β-catenin* depletion.^42^ These observations suggest that intercellular junctions are crucial in regulating HERS development, subsequently affecting the duration of tooth root development — a finding that is consistent with our results. Our study, for the first time, demonstrates that BMP9 signaling governs root odontoblast function by modulating cell proliferation and intercellular junctions within HERS, thereby influencing the overall developmental trajectory of molar roots. This regulatory role of *Bmp9* highlights its essential function in tooth root morphogenesis and paves the way for understanding dental tissue development.

HERS is directly associated with the formation of the apical foramen. Together with the dental papilla and dental follicle, HERS forms what is termed the developing apical complex, known for its high proliferative and mineralizing potential.^43^ Under typical conditions, HERS extends to create an epithelial septum. In later stages of root development, the epithelial septum's opening narrows as dentin and cementum are deposited, culminating in the formation of a narrow apical foramen. Studies have demonstrated that *Osr2-Cre*;*Ror2*^*fl/fl*^ mice exhibit reduced mesenchymal cell proliferation shortly after birth, which subsequently leads to HERS malformation.^44^ This phenomenon may involve the mesenchyme's role in forming a structural framework that supports HERS epithelial invagination.^45^ Based on these insights, we demonstrated that 4-week-old *Bmp9*-KO mice exhibited a significantly widened apical foramen compared with WT controls. This observation leads us to postulate that the structural abnormalities in HERS of *Bmp9*-KO mice may originate from dysregulated proliferative activity, compromising the organizational integrity of HERS. These proliferative changes could impair epithelial septum formation and ultimately result in incomplete closure of the apical foramen. This hypothesis aligns with the proliferative and structural dependencies of HERS in proper root development, highlighting *Bmp9*'s role in maintaining apical complex integrity and root morphology.

The loss of *Bmp9* reduces the proliferative capacity of HERS while also inhibiting odontoblast differentiation, secretion, and mineralization. Prior studies indicate that odontoblastic osterix (*Osx*)-conditional knockout mice display a root phenotype characterized by shortened and thin roots. Further investigation into this phenotype has revealed a complex regulatory network involving *Osx* expression, which is modulated by epithelial BMP signaling, mesenchymal Runt-related transcription factor 2 (*Runx2*) expression, and cellular phosphorylation levels.^46^ This evidence supports the broader role of BMP signaling in tooth root development through its influence on odontoblast regulation. Furthermore, research on B-box (*Bbx*) knockout mice shows slightly shorter mesial and distal molar roots compared with WT mice. *Bbx* appears to contribute to tooth root formation, with *Bbx* deficiency significantly decreasing *Dspp* expression, potentially due to its regulatory role on *Dspp*.^47^ These findings align with our study's results. However, as development progresses, compensatory mechanisms may partially mitigate these effects. By 8 weeks, for example, the apical foramen of the mandibular first molar in *Bmp9*-KO mice neared closure, resembling the closure pattern seen in WT mice, although root length remained shorter than in WT mice. This observation suggests a potential compensatory response that partially restores structural integrity despite persistent root shortening in *Bmp9*-deficient conditions.

In normal conditions, specialized intercellular junctions, such as desmosomes, gap junctions, and tight junctions, are present between adjacent odontoblasts, collectively forming junction complexes. Tight junctions are primarily located at the odontoblast tips in developing teeth, where they act as channels for intercellular signaling molecules, supporting secretion and mineralization processes. Deletion of *Zo-1* has been shown to disrupt mitotic spindle positioning in intestinal epithelial cells, inhibiting proliferation and promoting apoptosis.^48^ This evidence implies that *Zo-1* may directly influence cell proliferation, though the effects observed in our study suggest that *Zo-1* may function differently in root epithelial cells compared with odontoblasts. Further investigation is needed to clarify *Zo-1*'s specific roles within these cell types. In *Bmp9*-KO mice, the mandibular first molar root was notably shorter, the apical foramen was wider, and dentin thickness was reduced. This phenotype resembles that of dentinogenesis imperfecta type III (DI-III).^24^ DI is an autosomal dominant genetic condition marked by abnormal dentin formation.^49^ Further research into DI's pathogenesis and disease phenotypes has identified that dentin malformation linked to *Dspp* mutations is now classified as dentin hypoplasia. In cases of DI-III, affected teeth develop into “shell teeth” characterized by thin dentin, underdeveloped roots, and enlarged pulp chambers and root canals. Exposed and rapidly worn dentin leaves the pulp chamber vulnerable, often resulting in pulp exposure, alveolar abscesses, and premature tooth loss.^50^ While symptomatic treatment is commonly employed for DI-III, the extensive involvement of multiple teeth limits the overall effectiveness of these interventions.^51^

In summary, root development is a highly intricate process requiring coordinated interaction among various cell types and the precise regulation of multiple signaling pathways. This study demonstrates that in *Bmp9*-KO mice molars, odontoblast differentiation is impaired and delayed compared with WT mice. The loss of polarization in odontoblasts disrupts their secretory function, weakening DSPP secretion and subsequently delaying dentin mineralization. Furthermore, *Bmp9* ablation reduces the proliferative capacity of HERS cells and enhances intercellular tight junctions, thereby influencing root dentin formation and apical foramen closure. However, the role of *Bmp9* in enamel formation and periodontal ligament development has not been explored, presenting another important area for future research. Elucidating the molecular basis of tooth development in mice may provide insights into similar dental defects in patients and open potential pathways for therapeutic tooth regeneration strategies.

## Conclusions

This study compared developmental differences between *Bmp9*-KO mice and their WT littermate controls, revealing that *Bmp9*-KO mice displayed a shorter mandibular first molar root, a wider apical foramen, and thinner dentin. Mechanistically, *Bmp9* ablation impaired odontoblast differentiation in molars, delaying differentiation relative to WT mice. Disruption in odontoblast polarization affected their secretory capacity, weakening DSPP secretion, which led to delayed dentin mineralization, increased collagen type I deposition, and reduced root length. Further analyses demonstrated that *Bmp9* loss reduced cellular proliferation and enhanced intercellular junctions within the HERS, impeding root dentin formation and apical foramen closure. However, further research is required to explore the specific binding sites and modes of action of *Bmp9* in regulating HERS proliferation and intercellular connectivity integrity through specific signaling pathways.

## CRediT authorship contribution statement

**Chang Liu:** Writing – original draft, Visualization, Software, Methodology, Data curation, Conceptualization. **Hongyan Yuan:** Writing – original draft, Methodology, Investigation. **Jindie Huang:** Methodology, Data curation. **Shidian Ran:** Visualization, Methodology, Data curation. **Xiaorui Wei:** Methodology, Formal analysis. **Xingrui Yan:** Software, Methodology. **Linyu Xue:** Resources, Investigation. **Tong-Chuan He:** Writing – review & editing, Supervision, Resources, Project administration, Conceptualization. **Yuxin Zhang:** Formal analysis, Data curation. **Mengqin Gu:** Supervision, Resources. **Si Wu:** Supervision, Resources. **Fugui Zhang:** Supervision. **Wenping Luo:** Supervision, Resources, Methodology, Funding acquisition. **Hongmei Zhang:** Writing – review & editing, Supervision, Project administration, Funding acquisition.

## Funding

The reported work was supported by the 10.13039/501100001809National Natural Science Foundation of China (No. 82470977 to H.Z.; No. 32070539 to W.L.) and sponsored by the 10.13039/501100005230Natural Science Foundation of Chongqing, China (No. 2024ZYYB005 to H.Z.).

## Conflict of interests

Tong-Chuan he is the Editor-in-Chief of *Genes & Diseases*, he/she has no involvement in the peer-review of this article and has no access to information regarding its peer-review and other authors declared no conflict of interests.
